# Key Factors for Enhancing Home Care Workers’ Intention to Stay by Multiple-Criteria Decision Analysis

**DOI:** 10.3390/healthcare11050750

**Published:** 2023-03-03

**Authors:** Wei Hsu, Fang-Ping Shih

**Affiliations:** 1 Department of Health Care Management, National Taipei University of Nursing and Health Sciences, Taipei 112303, Taiwan; 2 Department of Human Resources—Personnel Section, Koo Foundation Sun Yat-Sen Cancer Center, Taipei 11259, Taiwan

**Keywords:** home care workers, intention to stay, multiple-criteria decision analysis (MCDA), decision-making trial and evaluation laboratory (DEMATEL), analytic network process (ANP)

## Abstract

The ageing population is increasing rapidly in Taiwan, where the ageing rate exceeds even that of Japan, the United States and France. The increase in the disabled population and the impact of the COVID-19 pandemic have resulted in an increase in the demand for long-term professional care, and the shortage of home care workers is one of the most important issues in the development of such care. This study explores the key factors that promote the retention of home care workers through multiple-criteria decision making (MCDM) to help managers of long-term care institutions retain home care talent. A hybrid model of multiple-criteria decision analysis (MCDA) combining Decision-Making Trial and Evaluation Laboratory (DEMATEL) and the analytic network process (ANP) was employed for relative analysis. Through literature discussion and interviews with experts, all factors that promote the retention and desire of home care workers were collected, and a hierarchical MCDM structure was constructed. Then, the hybrid MCDM model of DEMATEL and the ANP was used to analyze the questionnaire data of seven experts to evaluate the factor weights. According to the study results, the key direct factors are improving job satisfaction, supervisor leadership ability and respect, while salary and benefits are the indirect factor. This study uses the MCDA research method and establishes a framework by analyzing the facets and criteria of different factors to promote the retention of home care workers. The results will enable institutions to formulate relevant approaches to the key factors that promote the retention of domestic service personnel and to strengthen the intention of Taiwan’s home care workers to stay in the long-term care industry.

## 1. Introduction

It is estimated that the proportion of the elderly population in the European Union member states will continue to grow [1]. In the Italian social welfare system, hospital facilities for providing social health care to the elderly are limited. In conventional forms of old-age care, although systems and governance vary from region to region, care for the elderly can also be handled at the regional level by nonprofit institutions such as social cooperatives, which receive funding from municipalities and government departments to provide a range of regional services for the elderly [2]. With the increase in the ageing population and the general miniaturization of the family structure in Taiwan, the care needs of the disabled elderly have risen sharply. The proportion of the disabled population will also increase significantly, resulting in an increase in the demand and burden of long-term care that will aggravate the shortage of home care attendants.

The services of home care attendants range from companionship to life assistance, personal hygiene and cleaning, rehabilitation activities and housework. Since the content of home care services is quite diverse, there are life assistants and care attendants, both of which are important and indispensable roles for home care workers on the front line. Therefore, when implementing follow-up care plans for individual cases, the presence and retention of staff are important in regard to whether home care can be offered. Simply training and cultivating diverse and professional care service staff takes a large amount of time. In addition, the relationship and emotional challenges faced by home care attendants underscore the need for training [1]. The number of trainees and the turnover rate are key to the rapid growth of the home care service industry. In terms of the ageing rate in Taiwan, the number of people requiring long-term care services is rising, and with the limited government resources, long-term home care-related industries are facing changes in the social structure. The shortage of health care staff is a serious and chronic problem for long-term care in the 21st century, and research has shown that the reasons include disrespect, cultural influences, too much paperwork and changes in work style [3]. Due to the high turnover rate of resident staff and the decline in retention, the lack of staff will burden the management operations if it is not easy to find new resident staff and cultivate service staff talent. Long-term care institutions and the government must face this problem squarely; otherwise, it will lead to high labour costs, and the quality of care in overloaded long-term care institutions will be reduced. Thus, the issue of home care workers’ retention should be highlighted.

Home care services were provided by volunteers in the past, but in recent years, they have mainly been provided by hiring regular staff due to the long-term care policy. Taiwan is under the influence of socioeconomic changes, and people who participate in home care services have different personal backgrounds and service perceptions. Because the human capital of home care services is important for the long-term care of elderly and disabled individuals and home care attendants’ willingness to stay in their jobs can directly affect the quality of long-term care services, the retention of home care attendants must be evaluated and analyzed to understand their needs.

Numerous factors influence home care workers’ intention to stay. There have been many past studies on the factors influencing home care attendants’ willingness to stay, and most studies have used expert analysis methods, regression analysis methods and structural equations. Each method has advantages and disadvantages. For example, the expert research method is fast but overly subjective. The regression analysis method, under the assumption of a normal matrix, needs a large sample for research and discussion and cannot directly deal with the problem of category variable data. Multiple-criteria decision making (MCDM) is well suited to solve such problems. MCDM was designed to solve the uncertainty of planning (such as technology and risk) with multiple criteria and uses the structured method to stratify complex problems. The analytic hierarchy process (AHP) in MCDM has been used in different fields to discuss the brain drain and human capital care of enterprises, but the factors in the architecture may have interdependence or feedback relationships. If only the AHP method is considered, the causal relationships among the criteria cannot be considered, and the final overall priority weight is calculated using the average method. The weight of the final evaluation result will be higher or lower than the real situation, and there will be inaccuracies [4]. The analytic network process (ANP) can integrate the relationship among factors and AHP concepts. This study categorized the factors identified in the literature review to establish a preliminary research framework, considering the interaction among the criteria for promoting the retention of home care workers, which cannot be fully explained by the structure of the AHP. Therefore, it adopted the characteristics of solving the problem of internal dependence and external feedback of the cluster ANP.

To determine the relationships among the criteria of home care workers’ intention to stay, this study employed the powerful Decision-Making Trial and Evaluation Laboratory (DEMATEL) method, which uses expert questionnaires and graphical causal theory to understand the dependence and feedback relationship among the criteria and can be used to explore the core objectives and key criteria and to transform the degree of interaction between complex facets or clusters into characteristics of causality. DEMATEL can introduce the interaction impact weight among clusters into the ANP step and establish the relationship structure for the ANP method. To clarify the causal relationship and influence among the criteria, the MCDA model in this study combined DEMATEL and ANP to identify the key factors that promote home care attendants’ intention to stay.

Therefore, this study adopted the MCDA model, a combination of the DEMATEL and ANP methods, to establish a framework to determine the key factors that could significantly increase home care attendants’ willingness to stay. There are many work difficulties for home care workers in Taiwan, and some negative elements cause high turnover rates of home care employees, so workers in home care service institutions should be vaccinated. The findings of this study can provide managers of long-term care institutions with an understanding of the reasons for promoting the retention of front-line staff and a reference for formulating corresponding strategies or human resource management methods to reduce large organizational labour costs and loads. This study can improve the staffing problem of home care attendants and serve as a priority reference for long-term care institutions to improve the retention of home care attendants in the future. In addition, the findings may be helpful to provide concepts for policy makers to overcome the current global shortage of long-term care staff. This paper also enhances the human resources literature by demonstrating the key factors that promote home care workers’ intention to stay.

## 2. Literature Review

Due to the annually increasing needs of home care services and the lack of home care workers, many scholars have begun to pay attention to home care attendants’ intention to stay [5]. As home care providers face high turnover and problems in recruiting new employees, they should understand the factors that lead to the loss of home care staff in order to effectively provide better-quality home care [6]. In particular, employees need to feel valued for their contribution to the organization, and how they measure their willingness to stay is closely related to their motivation to stay. Employees’ retention can be measured in terms of burnout, organization and tendency to leave, and when employees subjectively believe that their needs are not met, they will inevitably not contribute to the organization’s goals or tasks and will eventually choose to leave [7]. Employees’ different perceptions of supervisors’ leadership styles can also lead to differences in their work attitudes [8]. For example, the more respectful managers are, the more engaged their subordinates are, and their willingness to stay then increases. The external retention motivation created by the organization effectively encourages caregivers to continue in the profession. Retention factors are particularly important for young or novice caregivers with precarious employment status. Many caregivers believe that internal retention motivations, such as commitment, conscience, interest in caregiving and responsibility, are the most important factors in convincing them to stay in the profession [9].

To identify the factors that strengthen and promote the retention intention of home care workers, the relevant literature was searched and collected, and the factors that scholars have found to promote employees retaining their intention to stay were comprehensively sorted. Then, appropriate evaluation criteria and sub-criteria were determined, and three aspects of the assessment criteria are summarized as “working environment”, “organization” and “future development”. The selected assessment components and criteria variables are explained in detail by means of a literature review.

### 2.1. Working Environment

Factors related to the desired working environment include relationship issues, i.e., problems with employers, supervisors, coworkers, doctors or subordinates. Regarding the aspect of the working environment, there may be bullying and harassment in the workplace. Emotional difficulties are composed of a lack of psychological support and excessive emotional needs. Problems associated with time pressure and quality of care include too many responsibilities, too many tasks, fear of making mistakes, making mistakes, not having enough time to care for patients, reduced conditions for caring for patients and time pressure leading to suboptimal work quality. In addition, employee independence was listed as a possible factor in the decision to leave. Work scheduling difficulties include unsatisfactory work plans, chaotic shifts, too many night shifts and too much overtime. Dissatisfaction with pay includes personal income needs, workload and low wage trends [10]. A number of scholars have proposed that the relevant factors of the working environment that promote employee retention include salary and benefits, flexible scheduling, low work pressure and workload, “good relationships with clients” and “avoiding workplace harassment”.

#### 2.1.1. Salary and Benefits

Salary and benefits refer to any form of financial compensation and welfare received by employees in a way that motivates their morale and improves their work efficiency so that employees with the required knowledge and ability can be retained [11]. Employees’ attitude towards benefits is usually an important predictor of their turnover rate, and salary satisfaction is quite important for employees. Some scholars have used a combination of customary domain and AHP to explore factors associated with the tendency to leave. The expert interviews revealed that the top five criteria are salary, health, bonus benefits, promotion opportunities and family care, with salary and benefits being among the top three criteria to which employees pay the most attention [11]. According to equity theory, when the staff perceive a mismatch between the pays/benefits ratio and their comparison to others’ ratios, they experience inequity. Associations were found between staff equity perceptions of benefits and intention to leave for staff working in services for individuals [12].

#### 2.1.2. Flexible Scheduling

Flexible scheduling refers to shift work, which usually refers to variable working hours, that is, working hours that are not fixed hours and schedules. In the face of changes in output demand, the shift system can adjust the amount of staffing in a timely manner and reduce labour costs, and if a flexible and coordinated shift and scheduling system is achieved, it can effectively promote employees’ intention to stay [13,14]. Home care attendants have a higher chance of working shifts or fixed night shifts, which not only increases work pressure but also reduces their willingness to stay. Some home care cases require 24-h care, so caregivers may be required to work shifts, and employment contracts between caregivers and institutions may affect and limit the shifts or scheduling patterns of caregivers.

#### 2.1.3. Low Work Pressure and Workload

Reducing work stress and load refers to improving employees’ overall health, increasing their performance and reducing burnout. For example, staff work independently or with a small number of colleagues, which increases their work pressure [15,16]. A study in Sweden using a cross-sectional survey design to explore the effects of work stress on dementia care specialists and other staff in home care services using t-tests and multiple linear regression analysis showed that due to the expected increase in the population of elderly people living at home, the need for extensive and complex health care is increasing, and there is a greater need for staff to work alone [17]. This strengthens their work pressure and generates more stress [17].

#### 2.1.4. Good Relationships with Clients

During the work of the home care attendant, there may be difficulties in interaction between the attendant and the client, and the family may also interfere with the provision of services or disagree with the home care attendant due to different views on the care, which may affect the progress of the care work. For home care attendants, communication problems with clients and families are one of the possible work difficulties. Salary and benefits, training, supervision and institutional support and relationship with the client are important factors in the retention of home care attendants [18]. If the home care attendant does not establish a good relationship with the client, the emotional problems reflected by the client may cause the attendant to reduce his or her intention to stay. Therefore, in the process of work, if attendants feel the respect of the client and the client’s family, like their relationship with the client, enjoy the feeling of being needed and expect to help make others’ lives better, their willingness to stay will be higher [18,19].

#### 2.1.5. Avoiding Workplace Harassment

Avoiding workplace harassment means that when a resident staff member is required to enter the home to provide services, when there are concerns about their personal safety in relation to a person of the opposite sex, such as sexual harassment, measures should be put in place to avoid problems [20]. The workplace of the home attendant is the area of the client’s life, and some activities or behaviors in the household’s private life may not be appropriate when the care attendant is present. One study of 1214 care workers found that 12.8% had experienced sexual assault and 27.6% had experienced sexual harassment and felt that their personal safety was compromised when dealing with issues such as sexual harassment [20].

### 2.2. Organization

An organization is a social entity that has a specific structure and a system of coordinated activities and exists to achieve a specific goal. Organizations are formed by relationships between people, and organizations already exist when people interact with each other to achieve goals [21]. In the connection between the organization and employees, employees’ identification with the organization plays an important role. This study synthesizes the past literature and proposes the relevant factors of organizations that promote employee retention. Therefore, the organizational assessment includes professional image, respect, sense of accomplishment, organizational commitment, job satisfaction and supervisor leadership style.

#### 2.2.1. Professional Image

Professional image refers to the beginning of the employee’s professional experience, which is gradually internalized in the formation of professional behavior. After the profession interacts with its environment, the public forms an image of the profession in its best state [22]. An Iranian study showed that the general public’s attitude towards the nursing profession is not positive. The lack of understanding of the professional image of patients and their families is another factor affecting caregivers, and the turnover rate of home care workers should be improved by improving working conditions and raising the social status and image of the profession [23]. At present, the public’s perception of care attendants is part of the impression of care workers, making it difficult for them to pay attention to their profession. On the one hand, the dignity of the workplace cannot be improved. On the other hand, because of the lack of career promotion channels, coupled with low salary levels and lack of labour condition protection, even if there are long-term care students who graduate and obtain a resident service license, their willingness to enter the long-term care industry is low, resulting in a shortage of front-line personnel. The serious shortage not only leads to the loss of long-term care staff at the grassroots level but also wastes investment in educational resources. Therefore, government agencies should strengthen the professional image of resident service workers, increase professional recognition and enable those who wish to participate in the long-term care industry to clearly understand the progression from home care workers to instructors and supervisors so that they can develop professional competence and qualifications.

#### 2.2.2. Respect

Past research has mentioned that caregiver self-improvement, effective communication and support from managers to caregivers, integration of organizational structures, improved working conditions and creation of caregiver cultural competencies can promote a respectful working environment and reduce the likelihood of staff departure. Current changes in social value systems and implicit social norms are considered determinants of respectful workplaces, while caregiver respect refers to showing respect for others, perceived respect for the environment and self-respect. Sociocultural factors are challenges that affect respect for the client and his or her family and other members of the care team [24].

#### 2.2.3. Sense of Accomplishment

Sense of accomplishment refers to a deep-seated motivation for individuals to engage in challenging tasks and improve their performance to achieve their goals [25]. In addition, increasing a personal sense of accomplishment can also reduce burnout and increase intention to stay [26]. High burnout and severe declines in job satisfaction and personal sense of accomplishment are obvious problems for caregivers in China, and improving the working environment may be an effective strategy for health care organizations to improve care outcomes [27].

#### 2.2.4. Organizational Commitment

Organizational commitment refers to the degree of individual recognition of and commitment to a particular organization, creating a work team with high performance and loyalty to the organization. This enables members to realize their potential and voluntarily accept the goals and values of the organization so that they will have a strong willingness to stay in order to effectively take advantage of the organization’s abundant human resources [28]. Managing and monitoring employee turnover is an important retention factor for organizations, as are determining appropriate criteria and minimizing negative consequences with effective solutions.

#### 2.2.5. Job Satisfaction

Job satisfaction refers to the employee’s feelings about the enterprise or aspects of it and is a subjective response to the work situation, including the individual’s physical and psychological satisfaction with the working environment and the work itself and the degree to which he or she likes (is satisfied with) or does not like (is dissatisfied with) his or her work. Expressing job satisfaction represents the extent to which an individual experiences happiness in an organizational context [29]. According to a cross-sectional Australian study, job satisfaction is the only important predictor of willingness to leave, so understanding and addressing job satisfaction is important. Understanding the experience of caregivers contributes to workforce development, supporting skilled caregivers in working within their area of practice, retaining experienced caregivers and supporting the recruitment of new caregivers [30]. Facility managers and human resources practitioners need to develop programs to increase caregivers’ job satisfaction, thereby fostering caregivers’ sense of responsibility [31] and increasing their retention. In addition, dissatisfaction with the opportunity to use competence and autonomy is caused by more responsibilities, high career challenges, not enough freedom of decision making, difficulty of work, too much routine work, etc. These problems will also affect retention factors, and the lack of improvement can decrease the chances of employee retention [10].

#### 2.2.6. Supervisor Leadership Style

Home care supervisor leadership refers to the two-way interaction between supervisors and staff, which is important for interpersonal relationships in the workplace. If supervisors do not identify their own emotional problems or recognize employees’ feelings, which can easily lead to the breakdown of interactive relationships, they cannot lead employees to reduce negative emotions to improve work results and prevent burnout and separation [32]. Good leadership is considered a superior model because it generates greater trust and motivation among subordinates. Caregivers experiencing work-related bullying and burnout are more likely to want to leave their jobs and careers, highlighting the importance of leadership management in preventing negative outcomes for employees and organizations [32]. Additionally, establishing strong, authentic leadership and management can help reduce caregiver burnout and turnover propensities [26]. The education and support function of the supervision system can reduce the impact of risk factors in the working environment and have a positive effect on the continuation of staff members’ careers; when the education, support and coordination functions of the supervisor are better executed, the willingness of the staff to stay will be higher [33].

### 2.3. Future Development

Each decision to leave is driven by identifiable factors, such as a desire to change careers, start a business, seek opportunities or switch companies. Due to its negative impact, employee turnover is a challenge for many companies. Increased employee turnover often reduces a company’s performance, especially for smaller companies with limited resources. When an employee resigns, the company must look for a replacement (high cost), which includes recruitment and training costs [34]. Therefore, the assessment of future employee development, including training programs, promotion opportunities and career development, is regarded as an important retention factor in the working environment of employees.

#### 2.3.1. Training Programs

Training programs refers to one of the ways an organization can persuade employees that the organization values them and that their development is to prepare for future jobs in addition to effectively performing current work. Training and development is also a radical change in employees’ knowledge, attitudes and skills to improve the quality and efficiency of their work, representing a significant investment in human capital [35,36,37]. Employee training programs affect employee turnover, and as management training increases, employees become less willing to leave [35]. To improve employee retention, companies should adopt appropriate career development policies, such as arranging training courses and seminars or providing incentives to learn new technologies to help employees overcome their fear of obsolescence and motivate them to contribute to the organization [7]. A Canadian study used a generalized estimation equation model to assess individual and organizational predictors of job satisfaction among allied health care providers and found that providing adequate education improved job satisfaction, reduced staff turnover and led to the provision of better-quality residential care [37].

#### 2.3.2. Promotion Opportunities

Opportunities for advancement mean higher job grades, higher salaries or greater responsibilities. As employees pursue a sense of professional competence, promotion reduces the link between under improvement and the intention to improve professional competence [38]. In particular, research has found that the chances of promotion within a company were significantly positively correlated with job satisfaction and retention, and promotion opportunities had a positive predictive effect on job satisfaction [39]. Promotion means an employee moves to a higher position relative to someone who does not. Employees can derive satisfaction not only from higher earnings than their peers but also from higher rankings. For managers, retention measures for resident service staff can achieve appropriate results by establishing a good promotion channel and training system. After the basic professional knowledge and skills of the staff are upgraded, individuals can be promoted or developed to have more specific professional abilities and work independently [40]. Additionally, career promotion can increase the expectations of the staff in the organization and their willingness to stay [41].

#### 2.3.3. Career Development

Career development refers to the continuous course of an individual’s work or career throughout his or her life and positively affects whether he or she can find other jobs in the future. A lack of management skills means that training opportunities and departmental objectives are not clearly communicated to staff; a lack of career coaching, poor retention strategies and inadequate promotion opportunities all contribute to declining career prospects [42]. The results of a cross-sectional questionnaire survey showed that career development and higher salaries ranked highest as factors to motivate retention, and incentive programs aligned with employees’ work-related and personal needs were developed to improve employee satisfaction and reduce turnover, thereby improving the quality of health care [43]. By combining the above factors that promote the retention of home care attendants, managers can first better understand the retention factors and then improve and increase the quality and continuity of home-based care services.

## 3. Research Methodology

In this study, the MCDM method was used to construct the key factors that promote the voluntary retention of home care attendants. Additionally, it was used to identify the causal relationship between key factors and their mutual causation by combining the DEMATEL and ANP methods to improve the reliability of identifying the factors that promote the voluntary retention of home care attendants. The data collection of this study was divided into two phases. The first stage was the DEMATEL stage, which mainly explored the interaction among the important factors that promote home care workers’ intention to stay. The second stage was the ANP stage, which evaluated the weight of each factor.

### 3.1. DEMATEL

DEMATEL was used to clarify whether the components and criteria of key factors that promote the retention of home care attendants are related or feedback; additionally, this method can be used to construct a structural composition of the analytical network program. DEMATEL in this MCDA model has the following four steps:

- STEP 1: Define the factors and measurement scale

The 3 facets and 14 criteria were selected through the literature review, and the first stage of the DEMATEL questionnaire was defined and designed. The content of the first part of the DEMATEL questionnaire contained basic information, including service unit, service title and service seniority. The second part contained a brief definition of the 14 criteria that are important factors in promoting the retention of home care workers; for example, the future environment component comprises the definitions of three programs, namely training programs, promotion opportunities and career development. The third part explored the interrelationship between key factors that promote the retention of home care attendants, so the experts were given five levels, 0~4, divided into the following responses: 0 is no impact, 1 is low impact, 2 is medium impact, 3 is high impact and 4 is very high impact. A total of 188 questions were included in the DEMATEL questionnaire.

In terms of data collection, this study began by e-mailing invitations to act as experts to chief executives who had managed a long-term care institution in Taiwan for more than three years. Four managers of long-term care institutions replied to the e-mail and voluntarily participated in March 2022. To improve the reliability of the home care attendant discretionary evaluation system and the construction of the MCDM model, this study extended the invitation to participate in the data collection to one long-term care academic professor (with more than 20 years of experience), one senior home care service supervisor (with more than 3 years of service) and one senior home care worker (with more than 5 years of service). Then, the seven experts were asked to complete the DEMATEL questionnaires.

- STEP 2: Calculate the direct/indirect relationship matrix

After the experts completed the questionnaire and judged the influence of the two criteria, the degree of influence between factors was determined, and the correlation between factors was expressed by a matrix (Equation (1)).

(1) $$X=\left[\begin{array}{cccc} 0 & x_{12} & \cdots & x_{1n}\\ x_{21} & \cdots & \cdots & x_{2n}\\ \vdots & \vdots & \ddots & \vdots \\ x_{n1} & x_{n2} & \cdots & 0 \end{array}\right]$$

Then, a normalized direct relationship matrix was calculated. There are two ways to calculate the normalized direct relationship matrix: one is to take the column vector and the largest as the normalization base, and the other is to take the vector and the largest of the column or columns as the normalization base. In this study, the column vector and the largest were used as the normalization base. The calculation formula is shown in Equation (2).

(2) $$\lambda =\frac{1}{\begin{array}{c} Max\\ 1\leq i\leq n \end{array}\left(\sum_{j=1}^{n}x_{ij}\right)}\;$$

The normalized direct correlation matrix *N* can be calculated by Equations (1) and (2), with the direct correlation matrix *X* multiplied by the lambda value (Equation (3)). After the normalized relationship matrix was obtained, the direct/indirect relationship matrix *T* was established using Equation (4).

(3) N=λX

(4) $$T=\underset{k\to \infty }{\lim}\left(N+N^{2}+\cdots +N^{k}\right)=N{\left(I-N\right)}^{-1}$$

Equations (5) and (6) were used to calculate the total intensity *D_i_* of the affected criteria and the total intensity of the affected *R_j_*.

(5) $$D_{i}=\overset{n}{\underset{j=1}{\sum }}t_{ij}\;\left(i=1,2,\cdots ,n\right)$$

(6) $$R_{j}=\overset{n}{\underset{i=1}{\sum }}t_{ij}\;\left(j=1,2,\cdots ,n\right)$$

- STEP 3: Calculate the prominence and relation

(*D_k_* + *R_k_*) is the prominence, and *k* = *i* = *j* =1, 2, ..., *n*, which represents the degree to which this factor is affected, according to which the core degree of factor *k* in all problems can be revealed. (*D_k_* – *R_k_*) is defined as the degree of causation, which indicates the degree of difference between the influence of this factor. According to this value, the degree of causality to which factor k belongs in all problems can be displayed, and if it is positive, the factor is biased towards the cause class. If it is a negative table, the factor is biased towards the result class.

- STEP 4: Establish thresholds and causal diagrams

To present a more significant causal relationship, this study calculated the values of the direct and indirect relationship matrices of each facet, determined whether there were values with lower correlation that needed to be deleted by setting a threshold value, and selected values greater than or equal to the threshold value. Thresholds were determined in the following ways. First, they were set on the basis of the recommendations of decision makers or jointly with multiple experts [44,45] without specifying the specific process of how to determine them. Second, the statistical mean [46,47] or median [48] of the elements in the total relationship matrix was the mainstay, but the average value was easily affected by the extreme value and lacked a theoretical basis. Although the median was not affected by extreme values, it still lacked sensitivity to the representativeness of skewed data and was too subjective for the causal relationship of systemic factors. Third, based on the statistical distribution, the threshold could be calculated using μ + σ or μ + 1.5σ [49,50], where μ and σ are the mean and standard deviation (SD) of the elements of the full relationship matrix, respectively. Essentially, the total relational matrix data do not necessarily follow a normal distribution and therefore may not be consistent with reality [51]. Furthermore, if the threshold is set too high, the relationship between the facets may not be distinguishable, and if it is too low, the relationship between the facets may be complicated so that the important inter-facet relationship cannot be revealed. After discussion with the experts, the threshold value of this study was set in the third quartile (Q3), which was suggested by the previous research [52]. With Q3 as the threshold, some factors may not have entered the ANP questionnaire when assessing the relative importance of the interdependence between various components and criteria.

Causality diagrams simplify the complex relationships between factors, show the influence of each factor on other factors, facilitate understanding of the topic to be studied and provide direction for subsequent discussions. When plotting the causal diagram, prominence (*D_k_* + *R_k_*) was taken as the horizontal axis and the relation (*D_k_* − *R_k_*) as the vertical axis. The coordinate points of the characteristics of the above factors formed a coordinate graphic. When (*D_k_* − *R_k_*) is positive, attribute k is classified as a cause class or influence group, and when (*D_k_* − *R_k_*) is negative, attribute k is classified as a result class or affected group. A higher (*D_k_* − *R_k_*) indicates that the attribute affects and is affected by other attributes to a greater extent. Depending on the coordinate position of the relation and prominence, attributes can be divided into the following four categories:

(a) Core region: The relation (*D_k_* − *R_k_*) is positive, the prominence (*D_k_* + *R_k_*) value is high, and the degree of influence of other characteristics plus the degree of influence of other characteristics and the sum of influence are strongly affected. However, the degree of influence on other characteristics is greater and is biased towards the cause class. The representative attribute is the cause class, which is the driver of the problem.

(b) Drive region: The relation (*D_k_* − *R_k_*) is positive, the prominence (*D_k_* + *R_k_*) is low, and the degree of influence of other characteristics is high, but the degree of total influence is low, which belongs to the quality characteristic factors of the cause of the low sum influence. This indicates that attributes are independent and can affect only a few other attributes.

(c) Affected region: The relation (*D_k_* − *R_k_*) is negative, the prominence (*D_k_* + *R_k_*) value is higher, the degree of influence of other quality characteristics is higher, and the degree of total influence is low. However, the sum of the two effects is highly influential, biased towards the quality characteristics of the outcome category. Representative properties are the core problem that needs to be solved, but because they are properties of the result class, they cannot be directly improved.

(d) Independent region: The relation (*D_k_* − *R_k_*) is negative, and the prominence (*D_k_* + *R_k_*) value is low, which is highly affected by other characteristics. However, the degree of sum influence is low, which belongs to the result quality characteristic factors affected by the low sum. This indicates that the attribute is independent, has a low correlation with other factors and is affected by only a few other attributes.

### 3.2. The Procedure of the Analytic Network Process (ANP)

In the second stage, the ANP is used to understand the relative importance of the evaluation system criteria and for the network hierarchy of factors that can improve the construction to promote the retention of home care attendants. Therefore, the network analysis procedure method is used to obtain the relative weight of the evaluation system criteria and the criteria. The ANP is derived from the AHP decision-making model, and its decision-making process is closer to people’s decision-making process.

The AHP, on the other hand, is limited by the fact that the criteria are independent of each other and there is no interaction between them, which may oversimplify the problem; thus, its assessment may be biased. In the ANP, feedback within clusters (internal dependence) and between clusters (external dependence) is allowed, the complex impact of interaction in human society can be more accurately expressed through feedback, and a more complete decision-making structure is the result. Elements in the cluster can be linked to other cluster elements according to the user’s need to investigate the process. The evaluation of decision problems using ANP includes the following three steps [53]:

- STEP 1: Establish the structure of the guidelines and their relationships

**Figure 1:**
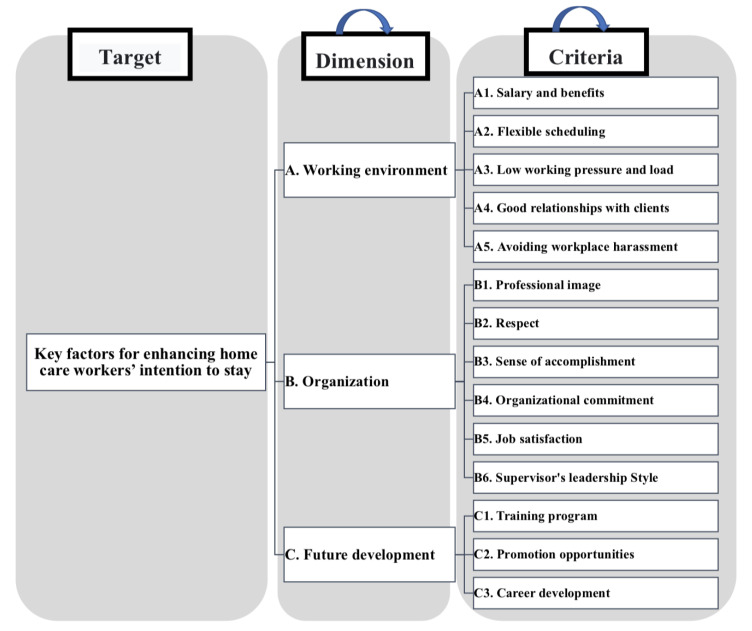
ANP-based model for enhancing home care workers’ intention of to stay.

Through the literature discussion and summarizing and formulating the important factors and criteria for promoting the retention of home care attendants, we established the structure of the guidelines and their relationships. We started the literature review using relevant key words, searching (“home care workers”, “home health aides”, “nursing aides”, “willingness to stay” and “intention to leave”). The literature records were identified through database searching (Web of Science and EBSCO). After removing duplicates and screening abstracts, we only kept the records with full text for eligibility. Then, we excluded the records which only discussed home care workers’ personal reasons (such as personal background, socioeconomic status, commuting convenience and stress from family conflict) because these factors could not be affected directly by the home care institutions and were not appropriate for the study aims. There were in total 19 records included, and 11 records were related to home care workers’ working environment, 10 were related to the organization factor and 7 were related to home care workers’ future development (some records mentioned more than two factors of home care workers’ intention to stay). There were three records mentioning “Salary and benefit (A1)”, one record mentioning “Flexible scheduling (A2)”, four records mentioning “Low working pressure and load (A3)”, four records mentioning “Good relationships with clients (A4)”, two records mentioning “Avoiding workplace harassment (A5)”, one record mentioning “Professional image (B1)”, two records mentioning “Respect (B2)”, two records mentioning “Sense of accomplishment (B3)”, one record mentioning “Organizational commitment (B4)”, two records mentioning “Job satisfaction (B5)”, three records mentioning “Supervisor’s leadership style (B6)”, two records mentioning “Training program (C1)”, three records mentioning “Promotion opportunities (C2)” and two records mentioning “Career development (C3)”. After integrating the included literature records, we built the structural chart of the factors which influence home care workers’ intention to stay. In total, 3 facets and 14 criteria of factors which influence home care workers’ intention to stay were included. The structural chart of the ANP criteria is shown in Figure 1. The MCDM research model was constructed through the DEMATEL and ANP method questionnaires to obtain appropriate factors to promote home care attendants’ intention to stay, so this structural chart was employed in both the DEMATEL and ANP stages.

- STEP 2: Make pairwise comparisons

This was the second stage of the questionnaire; the seven experts were asked to compare pairs between criteria with a total of 134 questions in the ANP questionnaire in May 2022. It included pairwise comparisons between dimensions and comparisons of criteria within dimension groups. There were nine levels (1–9) of standards to fill in: 1 is equal importance, 3 is slightly important, 5 is quite important, 7 is extremely important, 9 represents absolutely important, and 2, 4, 6, and 8 are intermediate values of adjacent scales. According to the seven experts’ scores of the relative importance of the criteria, the eigenvectors of each pairwise comparison matrix were finally calculated in the same way as in the analytical hierarchy program. Using Equation (7) to generate group priority matrix *A*, the factors of dependence were compared in pairs, and the priority weights were generated.

(7) $$A=\left[\begin{array}{cccc} 1 & A_{12} & \cdots & A_{1n}\\ 1/A_{12} & \cdots & \cdots & A_{2n}\\ \vdots & \vdots & \ddots & \vdots \\ 1/A_{1n} & 1/A_{2n} & \cdots & 1 \end{array}\right]$$

The ANP questionnaire was distributed to the same seven experts as the DEMATEL questionnaire, the same situation and background were discussed in depth and the research questions were analyzed more intensively. A total of seven questionnaires were completed. To comply with the consistency requirement, the CR value should be less than or equal to 0.1 when comparing the aspects and criteria of the evaluation system in compliance with conformity verification.

- STEP 3: Verify Conformity and Obtain Weights

After the ANP questionnaire was collected, the consistency verification procedure was carried out, and the consistency verification principle had to meet the principle of superiority and inferiority and the reproducibility of the strength relationship (transitivity) to ensure the validity of the questionnaire and the judgement of the experts who completed the questionnaire. Consistency verification is based on the consistency ratio of a pairwise comparison matrix; *C.R. = C.I./R.I.* where *C.I.* is consistency index and *R.I.* is random index. *C.R.* ≤ 0.1 was suggested to indicate that the degree of bias of decision makers in the judgement of the weights of each element when establishing the pairwise comparison matrix was still within an acceptable range, that is, there was consistency [54].

Then, a supermatrix structure was built, with all priority vectors placed in the supermatrix in the appropriate positions to produce an unweighted supermatrix. The criterion weights of the unweighted supermatrix were multiplied by the weights of the relevant facets so that the sum of the values of the rows was 1. The limit supermatrix was calculated from the weighted supermatrix. The weighted supermatrix was multiplied multiple times until the values of the columns were consistent and the final relative priority of each criterion could be obtained. The normalized limit supermatrix identified the relative weights of the group (criterion) and the element (alternative). The criterion with the higher weight indicated the criterion with the highest priority [55].

## 4. Study Results

This study mainly summarized the preliminary research framework through a literature review and categorized the aspects and criteria for promoting home care workers’ intention to stay. Seven experts and scholars were invited to complete the two questionnaires.

### 4.1. DEMATEL Results and Network Relationship Establishment

From the data collected by the DEMATEL questionnaire, the total influence matrix T of the three dimensions was obtained and is shown in Table 1. After filtering by threshold (Q3), the prominence (*D* + *R*) and the relation (*D* − *R*) calculated from the sum of rows and columns of the total influence matrix are shown in Table 1. Ranking by prominence (*D* + *R*), “Organization (B)” is the highest (21.424), followed by “Working environment (A)” and “Future development (C).” The results show that “Organization (B)” is the most visual component dimension to enhance home care workers’ intention to stay. Staff working in health organizations not only expect their basic physiological needs to be met but also gradually pay attention to the pursuit of self-image, sense of accomplishment and identity, and seek recognition of and respect for their professional skills. Meanwhile, regarding the values of the relation (*D* − *R*) from the table, “Work environment (A)” (1.347) is the “cause group” since the value is positive, while “Organization (B)” (−0.4 77) and “Future development (C)” (−0.871) are the “affected group”.

**Figure 2:**
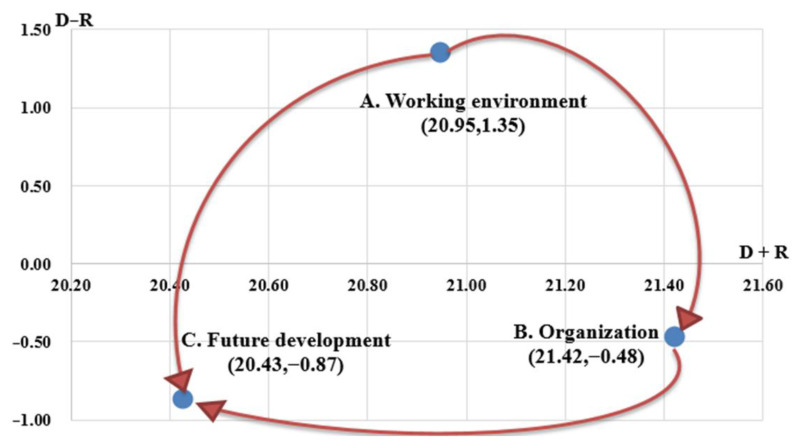
The impact digraph map of dimensions.

The information shown in Table 1 was plotted as a diagram of the influence relationship between the dimensions in Figure 2. The X-axis is the prominence (*D* + *R*), the Y-axis is the relation (*D* − *R*), the dimension of the “cause group” is at the top and the two dimensions of the “affected group” are at the bottom. The direction of the significant influences filtered through the threshold value of Q3 (3.658) is represented as the arrow. When the impact value was greater than or equal to the threshold value, the influence relationship was chosen and displayed by an arrow in the impact digraph map.

In addition, the impact digraph map of the dimensions shows that “Working environment (A)” is the “cause group” and “Organization (B)” and “Future development (C)” are the “affected group”, where the solid arrows point in the three one-way influence directions as follows: “Work environment (A)” affects “Organization (B)” and “Future development (C)”, and “Organization (B)” affects “Future development (C).” According to the prominence (*D* + *R*) and the relation (*D* − *R*), “Working environment (A)” was in the “core region” as the key influence factor and should be listed as the priority dimension. “Organization (B)” and “Future development (C)” belong to the “Affected region,” which means that their linkage could be improved when “Working environment (A)” is improved.

Similar to the dimension analysis process, the values of the same columns were summed to determine the average value, and the matrix was then normalized. Each element of the matrix was divided by the maximum column sum to obtain the normalized matrix and brought into the formula, and the threshold (Q3) was obtained by the total influence matrix *T* of the criteria in Table 2. The prominence (*D* + *R*) and the relation (*D* − *R*) were calculated from the sum of the rows and columns of the total influence matrix, as shown in Table 2. Table 2 shows the central degree of the prominence (*D* + *R*) of the criteria. According to the DEMATEL results, the top three criteria with the highest overall impact on the retention of home care workers were “Job satisfaction (B5)”, “Career development (C3)” and “Organizational commitment (B4)”.

Regarding the values of the relation (*D* − *R*) of the criteria, “Salary and benefits (A1)”, “Flexible scheduling (A2)”, “Training Program (C1)”, “Professional image (B1)”, “Avoiding workplace harassment (A5)” and “Supervisor Leadership (B6)” were all in the “cause group” since these criteria obtained positive values. “Building a good relationship with the client (A4)”, “Promotion opportunity (C2)”, “Respect (B2)”, “Career development (C3)”, “Organizational commitment (B4)”, “Job satisfaction (B5)”, “Sense of accomplishment (B3)” and “Reduce work stress and workload” (A3)” with negative values of the relation (*D* − *R*) were the “affected group”.

**Figure 3:**
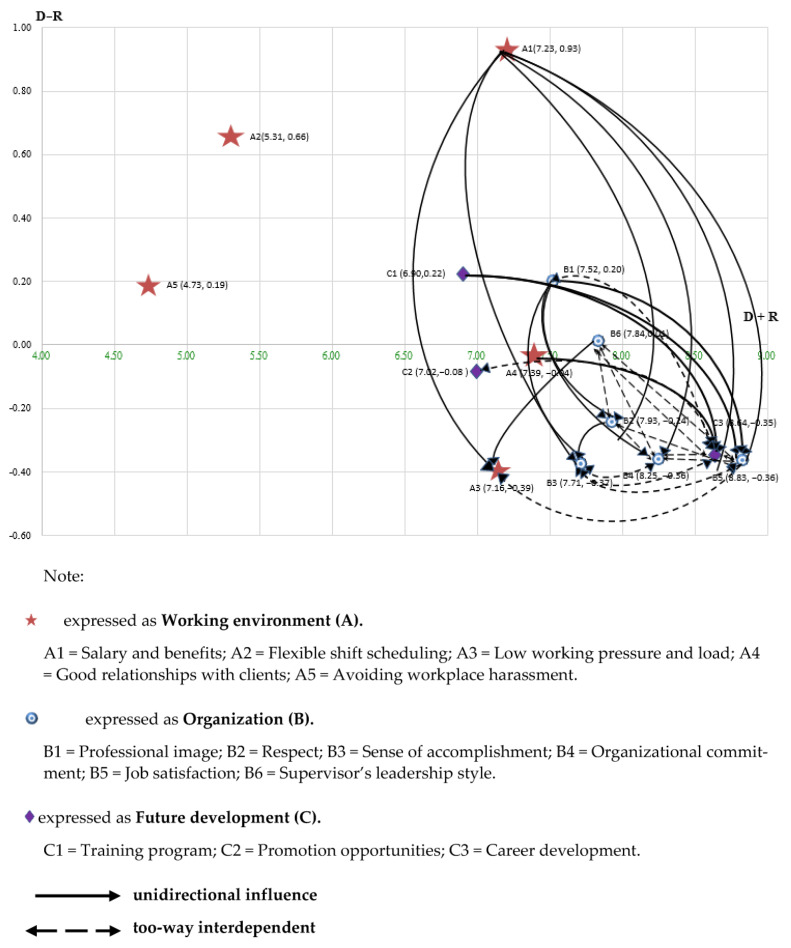
The impact digraph map of criteria.

The values of the prominence (*D* + *R*) and the relation (*D* − *R*) obtained in Table 2 were used to plot the impact digraph map to visualize the causal effects among the criteria, as shown in Figure 3. The X-axis is the prominence (*D* + *R*), and the Y-axis is the relation (*D* − *R*). The significant influences were filtered from the total-related matrix T of the criteria by setting the threshold (Q3 = 0.309). All the significant influences in which the influence value was greater than or equal to the threshold value were drawn as arrows to indicate the influence relationships among criteria to affect home care workers’ intention to stay.

In the impact digraph map of criteria (Figure 3), “Salary and benefits (A1)”, “Flexible scheduling (A2)”, “Avoiding workplace harassment (A5)”, “Professional image (B1)”, “Supervisor’s leadership style (B6)” and “Training program (C1)” were the “cause group”, and “Low work pressure and workload (A3)”, “Build a good relationship with the client (A4)”, “Respect (B2)”, “Sense of accomplishment (B3)”, “Organizational commitment (B4)”, “Job satisfaction (B5)”, “Promotion opportunities (C2)” and “Career Development (C3)” were the “affected group”. Among the criteria, the solid arrows indicate the one-way influence direction. The dotted arrows in Figure 3 show two-way influences.

The six criteria “Salary and benefits (A1)”, “Flexible scheduling (A2)”, “Avoiding workplace harassment (A5)”, “Professional image (B1)”, “Supervisor’s leadership style (B6)” and “Training program (C1)” were in the “core region” that addresses the key impact factors of home care workers’ intention to stay and should be prioritized. The seven criteria of “Reduction of work pressure and workload (A3)”, “Good relationships with clients (A4)”, “Respect (B2)”, “Sense of accomplishment (B3)”, “Organizational commitment (B4)”, “Job satisfaction (B5)”, “Promotion opportunities (C2)” and “Career development (C3)” were in the “affected region”, and their linkages would improve when those criteria in the “core region” were improved.

### 4.2. Analytic Network Process (ANP) Results

After the DEMATEL analysis to understand the complex causal relationships between the dimensions and the criteria, this section continued to employ the ANP method to calculate the quantitative weight of each criterion. Table 3 shows the results of the criteria weights. When the ANP weights were ranked, “Job satisfaction (B5)” was the highest (31.90%), followed by “Supervisor’s leadership style (B6)”, “Respect (B2)”, “Career development (C3)”, “Low work pressure and workload (A3)”, “Organizational commitment (B4)”, “Sense of accomplishment (B3)”, “Good relationships with clients (A4)”, “Promotion opportunities (C2)” and “Professional image (B1)”. The ANP weights of the remaining four criteria, “Salary and benefits (A1)”, “Flexible scheduling (A2)”, “Avoiding workplace harassment (A5)” and “Training program (C1)”, all converged to 0.0%. In addition, the weights of the criteria by the AHP method are displayed in Table 3, and the top three AHP weights were “Salary and benefits (A1)”, “Low work pressure and workload (A3)” and “Job satisfaction (B5)”.

### 4.3. Discussion

The prominence (*D* + *R*) values analyzed by DEMATEL indicated that the criterion “Job satisfaction (B5)” from the dimension “Organization (B)” had the highest overall impact; regarding the relation (*D* − *R*) values, the criterion “Salary and benefits package (A1)” in the dimension “Working environment (A)” had the highest net impact on home care workers’ intention to stay in the profession. The ANP method was used to determine the priority of importance of the factors that promote the retention of home care staff by considering the interdependent relationships conducted by DEMATEL simultaneously. The ANP results indicated that the top three key criteria for enhancing the retention of home care attendants were “Job satisfaction (B5)”, “Supervisor’s leadership style (B6)” and “Respect (B2)” in the dimension of “Organization (B)”. Overall, “Job satisfaction (B5)” had the highest weight and was the most important criterion.

According to past studies, employees who believe that managers genuinely care about their welfare will have higher job satisfaction, which in turn will reduce their willingness to leave [56]. Therefore, “Job satisfaction (B5)” was the main reason for willingness to stay and an important predictor of employees’ intention to stay. When a job does not meet employees’ needs, they might have the idea of leaving to find a better job. Being able to take care of and help clients so that they can have a sufficient sense of accomplishment and satisfaction and feel that they have contributed to society may strengthen their willingness to stay. The satisfaction of autonomy among home care workers has also often been mentioned in previous studies, and home workers often hope that their need for work autonomy can be satisfied.

Previous research has noted that there is a clear positive correlation between job satisfaction and career retention, and job satisfaction was the main determinant of employee retention [57] even during the COVID-19 pandemic [58,59]. The more satisfied employees are with their feelings about human interaction and their need for responsibility, the higher the likelihood of retention. If the organization fails to improve these retention factors to a satisfactory level, it may lead to departures, and these results are consistent with the literature. However, when home care workers are fully engaged in care services, they can not only significantly increase their job satisfaction and reduce their willingness to leave but also promote organizational citizenship behaviors [60], such as taking on more responsibilities and dedicating personal time to work. In addition, the retention of resident staff was positively correlated with an improvement in the quality of life of the residents under their care [38]. Therefore, if the job satisfaction of resident staff can be improved, it is actually indirectly helpful for residents and the organization as a whole.

Earlier research has shown that managers can treat others in a way that is related to their ability to achieve organizational goals, suggesting that the influence of managers or supervisors on employees comes mainly from their ability to create positive intrinsic motivation for staff, which sometimes affects employees more than money [61]. Therefore, the way supervisors treat staff is an important factor in promoting the voluntary behavior of resident staff. Different supervisor leadership styles produce different employee behaviors that can improve management performance, promote employee productivity and sustain team development. Therefore, adequate communication between home-based caregivers and their supervisor can reduce burnout and improve retention.

Home care workers themselves are becoming increasingly aware that care is a professional job and understanding the value and significance of this service field. However, some members of the public may not have a good understanding of home care work, and there is insufficient respect and recognition in their attitude towards home care workers. This will cause great frustration to home attendants, as when they receive unfriendly treatment from society, they will feel uncomfortable. This may even lead to passive, withdrawn behavior or self-questioning among home care staff.

Furthermore, although “Salary and benefits (A1)” is the highest net factor in promoting employee retention in the DEMATEL method, it is not the reason with the greatest weight in the ANP. For example, although managers can consider raising salaries or increasing annual bonuses, other institutions can increase their financial temptation in the same way. Therefore, it will not be mainly money considerations that will reduce their intention to stay. Since most home care workers are female, staff may differ in the weightings they give specific inputs or rewards, and pay was found to be less of an issue for female direct-care leavers [12]. “Salary and benefits (A1)” in the “core region” might not be a direct factor but influence home care workers’ intention to stay through the impact on other factors. “Job satisfaction (B5)”, “Supervisor treatment (B6)” and “Respect (B2)” can lead to satisfactory support and communication; therefore, these are the key factors in becoming a home care worker with a high level of caring. This also means that while the government is striving to increase the salary and benefits of home care attendants in order to retain human resources for home care, it may be more important to consider how to improve the respect for home care workers in society, meet the needs of the working environment and work itself, strengthen and improve the support system for supervision by agency heads, etc., to effectively retain the home care staff who already provide services in the practical field and attract young and new workers who are willing to devote themselves to the work of home care. If a manager or supervisor has strong communication with staff and can properly allocate time and labour, this has a positive impact on retention. This finding is also consistent with the literature, and leadership style is one of the key factors in promoting the retention of staff members.

To meet the long-term care staffing needs in Taiwan, the quality of home care services should be improved; the mutual circulation of resident staff, health care staff and the job market should be promoted; employment opportunities should be increased; and training courses should be integrated. The government organizes training courses every year, and at present, home care workers are required to attend the care worker training course to obtain a certificate of completion and to obtain a vocational technician certificate. Staff should regularly participate in training courses every year to maintain their expertise, which can indirectly enable them to feel confidence in their work and to show caring, patience and a professional image.

In summary, from the perspective of ANP weighting, “Job satisfaction (B5)”, “Supervisor’s leadership style (B6)” and “Respect (B2)” are the key factors in promoting the retention of home care workers. “Salary and benefits (A1)”, “Flexible scheduling (A2)”, “Avoiding workplace harassment (A5)”, Professional image (B1)” and “Training program (C1)” were not weighted in the ANP results, but in the DEMATEL analysis, those factors were in the “cause group”. This means that these factors might not directly affect home care workers’ intention to stay, but they might indirectly influence home care workers’ intention to stay through linkage effects. The current situation of home care workers in Taiwan is more flexible than that of hospital attendants in the past, and home care workers can arrange their own time and cases. Additionally, the organization allows more space for home care workers to choose their own cases to avoid sexual harassment in the workplace.

If we use the traditional AHP to understand the key factors that promote intention to stay, the findings reveal that the three most important criteria are “Salary and benefits (A1)”, “Low work pressure and workload (A3)” and “Job satisfaction (B5)”. However, through the MCDA model combined with DEMATEL and ANP, the three most criteria were “Job satisfaction (B5)”, “Supervisor’s leadership style (B6)” and “Respect (B2)”. This result indicates that if only the traditional AHP is considered, the results would be different. The reason is that the AHP assumed independency among the criteria and ignored the impact relationships among them. Although the hierarchy of the traditional AHP is linear, it does not consider that there may be feedback and dependence among the criteria, which can easily cause decision makers to make mistakes. In the MCDA model proposed in this study, the criteria are used in feedback and dependence simultaneously, so the results are more valuable and reduce the chance of misjudgment.

## 5. Conclusions and Recommendations for Future Research

With the development of the home care service industry, under the influence of the ageing society and the pandemic, society faces huge home care problems. Home care workers are the front line of home care staffing. They contact service recipients most frequently and are the most trusted by service recipients. In addition, clients may also face the dilemma of readapting and building a relationship of trust due to changes in caregivers if a hired home care attendant is a novice and is not familiar with the home care services. This might lead to a decline in the quality of care if it is impossible to recruit suitable home care workers quickly enough. This will cause work pressure and an excessive load on existing home care helpers. Although most caregivers faced new challenges in internal motivation to continue working during the COVID-19 pandemic, organizational rewards, financial incentives and the hope of changing employment status were all motivating factors for them to continue working [62]. This paper aimed to further explore the promotion of the retention of home care workers in the context of understanding the characteristics of home care work. Through various research and analysis methods and comprehensive results, this study identified the key factors that promote the retention of home care staff.

The results of the study show that organizations should focus on improving job satisfaction, supervisor leadership style and respect because job satisfaction is a true reflection of employees who can express themselves directly. For staff members, overall job satisfaction and the degree of feeling that affects their mood are related to promoting retention. Therefore, as an important retention factor, managers and human resources practitioners need to develop relevant programs to improve the job satisfaction of caregivers to cultivate their sense of responsibility [31]. They should pay more attention to the work of home attendants and have empathy. When home attendants encounter work setbacks, they can help calm their emotions and provide encouragement and support, provide positive affirmation with an attitude of appreciation and encouragement, publicly praise excellent home care attendants and publish information on websites or in institutional publications that can effectively enhance home care attendants’ work motivation and sense of belonging to the organization. In addition, if resident staff can share their care experience or care skills at regular meetings to enhance their sense of honor and achievement, when they engage in work, the clients they care for will also receive good service, thus achieving the win–win care goal of supply and demand.

Home care supervisors or managers should improve their leadership communication and training skills so that both the clients and the staff can feel fairly treated. Additionally, they should teach staff to take care of the practice to help make their work smoother and regularly take the initiative to investigate the work needs of staff to establish efficient communication channels and feedback channels, support their work based on the service staff’s response and create a friendly workplace (including supervision, a promotion system and festive event parties). This will encourage home care attendants to stay in their jobs for a long time.

When dispatching client services, home care institutions should consider the service route of the home attendants, assist them in applying for accident insurance, emphasize the service content to the clients and advocate the professionalism of the home attendants so that the public can improve their understanding of the roles and responsibilities of care attendants. This will shape the professional image of the care staff and enhance respect for them. This is a suggestion to increase their intention to stay, as it is necessary to improve this aspect to effectively achieve the retention of staff members.

Although “Salary and benefits (A1)” is the factor with the highest net impact on promoting the retention of staff in the DEMATEL, it is not the most weighted factor in the ANP results. Therefore, although “Salary and benefits (A1)” is the basic element of employee satisfaction, it is not the only factor. Additionally, the amount and quality of salary and benefits are no longer the most direct and main factors generally. Employers or managers think that employees mainly desire money and leave, but this finding reverses the stereotype that salary or pay is the only thing that workers care about when considering retention. This indicates that managers in the home care industry should pay attention to the differences from factors that have promoted the retention of employees in the past if they want to provide improved salary and benefits. The opinions of the resident staff about salary and benefits should be investigated regularly, the salary and benefits of the resident staff of different home care institutions should be compared so that the appropriate salary and benefits treatment can be given according to the situation of the institution. This result also coincides with the concept of equity theory. When providing reasonable salary and benefits, attention should be paid to the leadership and management of the supervisor and the due respect of the home care staff.

The hybrid MCDA model of DEMATEL and the ANP, compared with the AHP, is currently the most suitable method not only for determining the mutual influence relationship but also for effectively solving the problem of incompatibility and feedback between AHP aspects and criteria. If the combination of the DEMATEL and ANP methods is not considered, researchers may be unable to determine the truly important factors.

The purpose of this study is to understand the key factors of the success of home care attendants that are truly important to help managers of long-term care institutions retain home care talent. If home care institution managers can understand the important factors that promote the retention of home care staff, on the one hand, agency managers will be able to understand that home care staff want to stay in the institution, and on the other hand, they will be able to face problems or difficulties in the future and introduce practical and policy-related suggestions for future improvement. In this way, they can achieve the goal of effectively promoting the retention of staff.

This study mainly uses the MCDM research method to explore the factors that promote the retention of home care attendants. Although the causal influence and key factors can be understood, in the future, big data analysis might be used to consider home care workers as the research object and to verify the relationships among the factors that promote the retention of home care attendants. This would resolve the limitations of the MCDA model, such as the few expert observations and the inability to verify the mediation or moderation effect analysis. In addition, it is suggested that in the future, the scope of research could be extended to other long-term care industries (e.g., community or institutional) to compare whether the key factors of care workers’ intention to stay are similar. Finally, this study ignores the ambiguity of decision-makers’ judgements, so future research can apply the concept of fuzzy theory for in-depth discussion so that researchers can obtain objective information, opinions and insights through the independent judgement of multiple experts in the process of information collection. In this way, agency managers could gain a deeper understanding of the different views of staff retention in their organizations, and the depth and breadth of research could be expanded to achieve the goal of promoting the retention of employees.

In the past, there has been no study using the DEMATEL and ANP methods to discuss the factors that promote the retention of home attendants. This research method solves the problem of stratification in a structured way to quantitatively determine the problem of decomposition in different aspects and hierarchies. It provides an overall final evaluation score and judgement of the criteria to construct an MCDM structure of the factors that promote the retention of home care workers. In practice, this study provides managers of home care institutions with a better understanding of ways to promote the retention of home care workers and allows them to consider coping methods based on the factors generated by the results of this study. Managers can implement activities and plans to increase the retention of home care staff in their institutions. Therefore, this study provides practical ideas for promoting the retention of home care workers and benefiting people with home care needs, the entire home care industry and government agencies. The future ageing problem is a major challenge that the country must face, and how to cultivate and retain exceptional home care talents is an important issue.

## Figures and Tables

**Table 1:** The total-related matrix T of dimensions (threshold value ≥3.658).

Dimension	A	B	C	*D*	*R*	*D* + *R*	*D* − *R*	Ranking Result	
								*D* + *R*	*D* − *R*
**A**	**3.272**	***4.003***	***3.873***	**11.149**	**9.801**	**20.950**	1.347	2	1
**B**	3.382	3.433	***3.658***	10.474	10.951	21.424	−0.477	1	2
**C**	3.147	3.514	3.118	9.779	10.650	20.429	−0.871	3	3

Note: A = Working environment; B = Organization; C = Future development. *D* + *R* is the prominence and *D* − *R* is the relation.

**Table 2:** The total-related matrix T of the criteria (threshold value ≥0.309).

Criterion	A1	A2	A3	A4	A5	B1	B2	B3	B4	B5	B6	C1	C2	C3	*D*	*R*	*D* + *R*	*D* − *R*	Ranking Result	
																			*D* + *R*	*D* − *R*
**A1**	0.202	0.205	***0.310***	0.287	0.169	0.300	***0.317***	***0.333***	***0.355***	***0.376***	0.303	0.274	0.282	***0.367***	4.080	3.149	7.229	0.930	9	1
**A2**	0.193	0.111	0.252	0.223	0.134	0.189	0.226	0.208	0.265	0.295	0.243	0.188	0.195	0.263	2.986	2.327	5.314	0.659	13	2
**A3**	0.217	0.174	0.201	0.253	0.155	0.226	0.249	0.248	0.298	***0.329***	0.270	0.220	0.235	0.308	3.383	3.775	7.158	−0.393	10	14
**A4**	0.220	0.176	0.286	0.214	0.180	0.276	0.308	0.296	0.300	***0.337***	0.282	0.221	0.254	***0.325***	3.675	3.719	7.394	−0.043	8	7
**A5**	0.140	0.108	0.188	0.197	0.088	0.181	0.207	0.179	0.212	0.236	0.191	0.157	0.159	0.219	2.462	2.271	4.734	0.191	14	5
**B1**	0.253	0.165	0.263	0.291	0.190	0.223	***0.328***	***0.323***	***0.316***	***0.343***	0.296	0.262	0.272	***0.338***	3.861	3.660	7.522	0.201	7	4
**B2**	0.222	0.162	0.269	0.294	0.197	0.298	0.248	***0.321***	***0.331***	***0.351***	***0.312***	0.244	0.258	***0.340***	3.845	4.088	7.933	−0.243	4	9
**B3**	0.231	0.156	0.266	0.273	0.152	0.268	0.303	0.235	***0.314***	***0.336***	0.285	0.246	0.269	***0.335***	3.669	4.044	7.713	−0.375	6	13
**B4**	0.248	0.193	0.295	0.285	0.169	0.289	***0.312***	***0.317***	0.265	***0.349***	***0.311***	0.270	0.290	***0.350***	3.945	4.305	8.250	−0.360	3	11
**B5**	0.265	0.194	***0.326***	***0.325***	0.182	0.287	***0.341***	***0.352***	***0.365***	0.304	***0.336***	0.278	0.300	***0.381***	4.235	4.599	8.834	−0.364	1	12
**B6**	0.229	0.192	***0.311***	0.285	0.186	0.284	***0.333***	0.308	***0.340***	***0.351***	0.240	0.251	0.268	***0.345***	3.924	3.911	7.835	0.013	5	6
**C1**	0.239	0.152	0.259	0.266	0.169	0.272	0.285	0.287	0.292	***0.312***	0.267	0.187	0.259	***0.317***	3.562	3.340	6.902	0.222	12	3
**C2**	0.222	0.144	0.250	0.238	0.141	0.257	0.289	0.292	0.300	0.308	0.262	0.250	0.195	***0.318***	3.466	3.550	7.016	−0.085	11	8
**C3**	0.269	0.193	0.300	0.287	0.159	***0.312***	***0.342***	***0.345***	***0.352***	***0.373***	***0.314***	0.292	***0.315***	0.292	4.144	4.498	8.642	−0.353	2	10

Note: Working environment (A): A1 = Salary and benefits; A2 = Flexible shift scheduling; A3 = Low working pressure and load; A4 = Good relationships with clients; A5 = Avoiding workplace harassment. Organization (B): B1 = Professional Image; B2 = Respect; B3 = Sense of accomplishment; B4 = Organizational Commitment; B5 = Job Satisfaction; B6 = Supervisor’s leadership Style. Future Development (C): C1 = Training Program; C2 = Promotion Opportunities; C3 = Career Development. *D* + *R* is the prominence and *D* − *R* is the relation.

**Table 3:** Comparison of critical success factors weights in AHP and ANP.

Dimension	Criterion	AHP Weights (%)	ANP Weights (%)	AHP (Ranking)	ANP (Ranking)
Working environment (A)	A1 Salary and benefits	33.58	0.00	1	11
	A2 Flexible shift scheduling	7.80	0.00	4	12
	A3 Low working pressure and load	10.72	9.99	2	5
	A4 Good relationships with clients	5.76	3.40	6	8
	A5 Avoiding workplace harassment	6.27	0.00	5	14
Organization (B)	B1 Professional image	1.53	0.76	14	10
	B2 Respect	3.10	11.61	9	3
	B3 Sense of accomplishment	2.95	6.77	11	7
	B4 Organizational commitment	2.30	8.08	13	6
	B5 Job satisfaction	9.68	31.90	3	1
	B6 Supervisor’s leadership style	5.58	15.40	7	2
Future development (C)	C1 Training program	5.14	0.00	8	13
	C2 Promotion opportunities	3.10	1.85	10	9
	C3 Career development	2.49	10.24	12	4

## Data Availability

Not applicable.

## References

[1] Gazzaroli D., D’Angelo C., Corvino C. (2020). Home-Care Workers’ Representations of Their Role and Competences: A Diaphanous Profession. Front. Psychol..

[2] Scrinzi F. (2019). Beyond ‘women’s work’: Gender, ethnicity, and the management of paid care work in nonprofit domiciliary services in Italy. J. Immigr. Refugee Stud..

[3] Barney S.M. (2002). The nursing shortage: Why is it happening?. J. Healthc. Manag..

[4] Ou Yang Y.P., Shieh H.M., Leu J.D., Tzeng G.H. (2008). A novel hybrid MCDM model combined with DEMATEL and ANP with applications. Int. J. Oper. Res..

[5] Steinmetz S., de Vries D.H., Tijdens K.G. (2014). Should I stay or should I go? The impact of working time and wages on retention in the health workforce. Hum. Resour. Health.

[6] Carpenter M.L., Blaskewicz Boron J., Beadle J., Potter J.F. (2021). Understanding Influential Factors in Turnover within the Home Care Workforce. Home Health Care Manag. Pract..

[7] Mak B.L., Sockel H. (2001). A confirmatory factor analysis of IS employee motivation and retention. Inf. Manag..

[8] Testa M.R., Sipe L. (2012). Service-Leadership Competencies for Hospitality and Tourism Management. Int. J. Hosp. Manag..

[9] Jeong S.A., Kim J. (2022). Factors influencing nurses’ intention to care for patients with COVID-19: Focusing on positive psychological capital and nursing professionalism. PLoS ONE.

[10] Estryn-Behar M., Van Der Heijden B.I., Fry C., Hasselhorn H.M. (2010). Longitudinal analysis of personal and work-related factors associated with turnover among nurses. Nurs. Res..

[11] Carraher S.M. (2011). Turnover prediction using attitudes towards benefits, pay, and pay satisfaction among employees and entrepreneurs in Estonia, Latvia, and Lithuania. Balt. J. Manag..

[12] Disley P., Hatton C., Dagnan D. (2009). Applying equity theory to staff working with individuals with intellectual disabilities. J. Intellect. Dev. Disabil..

[13] Teclaw R., Osatuke K. (2015). Nurse perceptions of workplace environment: Differences across shifts. J. Nurs. Manag..

[14] Li X., Zhang Y., Yan D., Wen F., Zhang Y. (2020). Nurses’ intention to stay: The impact of perceived organizational support, job control and Job satisfaction. J. Adv. Nurs..

[15] Mirzaei A., Rezakhani Moghaddam H., Habibi Soola A. (2021). Identifying the predictors of turnover intention based on psychosocial factors of nurses during the COVID-19 outbreak. Nurs. Open.

[16] Bratt C., Gautun H. (2018). Should I stay or should I go? Nurses’ intensions to leave nursing homes and home nursing. J. Nurs. Manag..

[17] Sandberg L., Borell L., Edvardsson D., Rosenberg L., Boström A.M. (2018). Job strain: A cross-sectional survey of dementia care specialists and other staff in Swedish home care services. J. Multidiscip. Healthc..

[18] Faul A.C., Schapmire T.J., D’Ambrosio J., Feaster D., Oak C.S., Farley A. (2010). Promoting Sustainability in Frontline Home Care Aides: Understanding Factors Affecting Job Retention in the Home Care Workforce. Home Health Care Manag. Pract..

[19] Tourangeau A., Patterson E., Rowe A., Saari M., Thomson H., MacDonald G., Cranley L., Squires M. (2014). Factors influencing home care nurse intention to remain employed. J. Nurs. Manag..

[20] Hanson G.C., Perrin N.A., Moss H., Laharnar N., Glass N. (2015). Workplace Violence against Homecare Workers and Its Relationship with Workers Health Outcomes: ACross-Sectional Study. BioMed Cent. Public Health.

[21] Daft R.L. (2015). Organization Theory and Design.

[22] Ngwenya V.C. (2021). Job performance: Working conditions of urban teachers in Zimbabwean schools. SA J. Hum. Resour. Manag..

[23] Alilu L., Zamanzadeh V., Valizadeh L., Habibzadeh H., Gillespie M. (2017). A Grounded theory study of the intention of nurses to leave the profession1. Rev. Lat.-Am. Enferm..

[24] Nouri A., Sanagoo A., Jouybari L., Taleghani F. (2021). Contextual barriers of Respectful workplace in nursing: A focused ethnography. Iran. J. Nurs. Midwifery Res..

[25] Fodor E.M., Schultheiss O.C., Brunstein J.C. (2010). Power Motivation.

[26] Na S.Y., Park H. (2019). The effect of nurse’s emotional labor on turnover intention: Mediation effect of burnout and moderated mediation effect of authentic leadership. J. Korean Acad. Nurs..

[27] Zhang L.F., You L.M., Liu K., Zheng J., Fang J.B., Lu M.M., Bu X.Q. (2014). The association of Chinese hospital work environment with nurse burnout, Job satisfaction, and intention to leave. Nurs. Outlook.

[28] Thompson J.E. (1989). An alternative control model for event studies. J. Bus. Financ. Account..

[29] Luz C.M.D.R., de Paula S.L., de Oliveira L.M.B. (2018). Organizational commitment, Job satisfaction and their possible influences on intent to turnover. Revista Gestão.

[30] Halcomb E., Bird S. (2020). Job satisfaction and Career Intention of Australian General Practice Nurses: A Cross: Sectional Survey. J. Nurs. Scholarsh..

[31] Gebregziabher D., Berhanie E., Berihu H., Belstie A., Teklay G. (2020). The relationship between Job satisfaction and turnover intention among nurses in Axum comprehensive and specialized hospital Tigray, Ethiopia. BioMed Cent. Nurs..

[32] Laschinger H.K.S., Fida R. (2014). A time-lagged analysis of the effect of authentic leadership on workplace bullying, burnout, and occupational turnover intentions. Eur. J. Work. Organ. Psychol..

[33] McCaughey D., Kim J., McGhan G., Jablonski R., Brannon D. (2010). Who Needs Caring? We Do! Workplace Injury and Its Effect on Home Health Aides. Acad. Manag. Annu. Meet. Proc..

[34] Memon M.A., Sallaeh R., Rosli Baharom M.N., Nordin S.M., Ting H. (2017). The relationship between training satisfaction, organisational citizenship behaviour, and turnover intention: A PLS-SEM approach. J. Organ. Eff. People Perform..

[35] Malek K., Kline S.F., DiPietro R. (2018). The impact of manager training on employee turnover intentions. J. Hosp. Tour. Insights.

[36] Ogbonnaya C., Tillman C.J., Gonzalez K. (2018). Perceived organizational support in health care: The importance of teamwork and training for employee well-being and patient satisfaction. Group Organ. Manag..

[37] Aloisio L.D., Gifford W.A., McGilton K.S., Lalonde M., Estabrooks C.A., Squires J.E. (2018). Individual and organizational predictors of allied healthcare providers’ Job satisfaction in residential long-term care. BioMed Cent. Health Serv. Res..

[38] Rajamohan S., Porock D., Chang Y.P. (2019). Understanding the relationship between staff and Job satisfaction, stress, turnover, and staff outcomes in the person-centered care nursing home arena. J. Nurs. Scholarsh..

[39] Castle N.G., Degenholtz H., Rosen J. (2006). Determinants of staff Job satisfaction of caregivers in two nursing homes in Pennsylvania. BMC Health Serv. Res..

[40] Pierson M.A., Liggett C., Moore K.S. (2010). Twenty years of experience with a clinical ladder: A tool for professional growth, evidence-based practice, recruitment, and retention. J. Contin. Educ. Nurs..

[41] Kosteas V.D. (2011). Job satisfaction and promotions. Ind. Relat. A J. Econ. Soc..

[42] Erasmus B.J. (2020). Perceptions of administrative staff on career advancement realities at a South African university. Manag.-J. Contemp. Manag. Issues.

[43] Al-Qathmi A., Zedan H. (2021). The effect of incentive management system on turnover rate, Job satisfaction and motivation of medical laboratory technologists. Health Serv. Res. Manag. Epidemiol..

[44] Hsu C.C. (2012). Evaluation criteria for blog design and analysis of causal relationships using factor analysis and DEMATEL. Expert Syst. Appl..

[45] Wang T.C. (2012). The interactive trade decision-making research: An application case of novel hybrid MCDM model. Econ. Model..

[46] Cebi S. (2013). Determining importance degrees of website design parameters based on interactions and types of websites. Decis. Support Syst..

[47] Horng J.S., Liu C.H., Chou S.F., Tsai C.Y. (2013). Creativity as a critical criterion for future restaurant space design: Developing a novel model with DEMATEL application. Int. J. Hosp. Manag..

[48] Bai C., Sarkis J. (2013). A grey-based DEMATEL model for evaluating business process management critical success factors. Int. J. Prod. Econ..

[49] Rajesh R., Ravi V. (2015). Modeling enablers of supply chain risk mitigation in electronic supply chains: A Grey–DEMATEL approach. Comput. Ind. Eng..

[50] Tzeng G.H., Huang C.Y. (2012). Combined DEMATEL technique with hybrid MCDM methods for creating the aspired intelligent global manufacturing & logistics systems. Ann. Oper. Res..

[51] Chen J.K. (2022). Multi-layer hierarchical DEMATEL method: Analysis of soft factors in TQM practice. J. Qual..

[52] Bolaños R., Fontela E., Nenclares A., Pastor P. (2005). Using interpretive structural modelling in strategic decision: Making groups. Manag. Decis..

[53] Meade L.M., Sarkis J.J.I.J. (1999). Analyzing organizational project alternatives for agile manufacturing processes: An analytical network approach. Int. J. Prod. Res..

[54] Saaty T.L. (1996). Decision Making with Dependence and Feedback: The Analytic Network Process.

[55] Yang C.H., Hsu W., Wu Y.L. (2022). A hybrid multiple-criteria decision portfolio with the resource constraints model of a smart healthcare management system for public medical centers. Socio-Econ. Plan. Sci..

[56] Babakus E., Yavas U., Karatepe O.M., Avci T. (2003). The effect of management commitment to service quality on employees’ affective and performance outcomes. J. Acad. Mark. Sci..

[57] McCreary D.D.J. (2020). Home health nursing Job satisfaction and retention: Meeting the growing need for home health nurses. Nurs. Clin..

[58] Nemțeanu M.S., Dinu V., Dabija D.C. (2021). Job insecurity, job instability and job satisfaction in the context of COVID-19 pandemic. J. Compet..

[59] Nemțeanu M.S., Dinu V., Pop R.A., Dabija D.C. (2022). Predicting job satisfaction and work engagement behavior in the COVID-19 pandemic: A conservation of resources theory approach. E&M Econ. Manag..

[60] Hsu W., Yang F.C. (2022). Factors associated with home health aides’ turnover intention and organizational citizenship behavior in long-term care services. Healthcare.

[61] Zebral L.P. (2017). The influence of leadership and payment for performance on individual performance. J. Appl. Leadersh. Manag..

[62] Cohen S., Nica E. (2021). COVID-19 pandemic-related emotional anxiety, perceived risk of infection, and acute depression among primary care providers. Psychosociological Issues Hum. Resour. Manag..

